# Resolutions of Respect, Dr. George C. Brown

**Published:** 1897-03

**Authors:** 


### Obituary.

### RESOLUTIONS OF RESPECT, DR. GEORGE C. BROWN.

The following resolutions were passed at a special meeting of the New Jersey State Dental Society, Friday evening, February 5, 1897, at Newark.

Whereas, It has pleased Almighty God to remove suddenly from the midst of his active labors our fellow-member and Treasurer, Dr. George C. Brown:

*Resolved*, That this society hereby desires to place upon record their high appreciation of his manly Christian character and of his high professional standing, as well as his genial and pleasant presence.

*Resolved*, That we mourn his death as a personal as well as professional loss, and hereby desire to testify of our high appreciation of his efforts he always and at all times gave to the profession of his adoption, and his devoted activity to the advancement of its welfare.

*Resolved*, That we mourn with his afflicted family, and extend to them in this hour of trial our sympathies.

*Resolved*, That a copy of this resolution be sent to his family and be placed upon the minutes of our society, and be published in the journals devoted to dentistry.

Signed, C. S. Stockton,

F. C. Barlow,

J. Allen Osmun,

*Chairman.*

